# Comprehensive Review of Cardiovascular Disease Risk in Nonalcoholic Fatty Liver Disease

**DOI:** 10.3390/jcdd9120419

**Published:** 2022-11-26

**Authors:** Kevan Josloff, Jacob Beiriger, Adnan Khan, Richard J. Gawel, Richard S. Kirby, Aaron D. Kendrick, Abhinav K. Rao, Roy X. Wang, Michelle M. Schafer, Margaret E. Pearce, Kashyap Chauhan, Yash B. Shah, Gregary D. Marhefka, Dina Halegoua-DeMarzio

**Affiliations:** 1 Sidney Kimmel Medical College, Thomas Jefferson University Hospital, Philadelphia, PA 19107, USA; 2 Department of Internal Medicine, Thomas Jefferson University Hospital, Philadelphia, PA 19107, USA; 3 Department of Internal Medicine, Division of Cardiology, Thomas Jefferson University Hospital, Philadelphia, PA 19107, USA; 4 Department of Internal Medicine, Division of Gastroenterology & Hepatology, Thomas Jefferson University Hospital, Philadelphia, PA 19107, USA

**Keywords:** NAFLD, CVD, MAFLD

## Abstract

Nonalcoholic Fatty Liver Disease (NAFLD) is a growing global phenomenon, and its damaging effects in terms of cardiovascular disease (CVD) risk are becoming more apparent. NAFLD is estimated to affect around one quarter of the world population and is often comorbid with other metabolic disorders including diabetes mellitus, hypertension, coronary artery disease, and metabolic syndrome. In this review, we examine the current evidence describing the many ways that NAFLD itself increases CVD risk. We also discuss the emerging and complex biochemical relationship between NAFLD and its common comorbid conditions, and how they coalesce to increase CVD risk. With NAFLD’s rising prevalence and deleterious effects on the cardiovascular system, a complete understanding of the disease must be undertaken, as well as effective strategies to prevent and treat its common comorbid conditions.

## 1. Introduction

Nonalcoholic fatty liver disease (NAFLD) is a highly prevalent and potentially life-threatening illness that has become the world’s most common chronic liver disease, affecting more than 1 in 4 people globally [1]. The prevalence of NAFLD exists in Africa (14%), Europe (23%), United States (24%), Asia (27%), South America (31%), and the Middle East (32%) [2]. NAFLD currently exists as the second most common indication for liver transplant and may increase hepatic and non-hepatic morbidity and mortality [3]. There is evidence that NAFLD has a causative relationship with metabolic syndromes [4], and a surplus of research reveals that NAFLD and nonalcoholic steatohepatitis (NASH) are linked to atherosclerosis and major cardiovascular events [5,6]. A meta-analysis of 27 cross sectional studies reported that there exists an association between markers of atherosclerosis and NAFLD [5]. Furthermore, this meta-analysis illustrated increased arterial stiffness indexes, carotid intimal-medial thickness, and coronary artery calcification scores [5]. Another meta-analysis incorporated 16 observational studies in literature and concluded that NAFLD demonstrated an increased risk of cardiovascular morbidity and mortality ([OR] 1.64, 95% CI 1.26–2.13) and a higher risk of CVD in severe NAFLD (OR 2.58; 1.78–3.75) [4]. On the other hand, more recent literature debates there is no significant association between NAFLD and CVD mortality incorporating 14 studies and 498,501 subjects [7]. A significant need exists for further research to investigate the relationships between NAFLD and specific cardiac complications, including ASCVD risk, heart failure, arrhythmias, HTN, CAD/atherosclerosis, and MI.

Metabolic syndrome and subsequent obesity, diabetes, and hypertension have been considered the most indicative of a pathophysiological association of mortality between NAFLD and CVD. It is important to make a note that NAFLD is present in normal body mass index (BMI) patients as well—where non-obese is defined as BMI < 25 kg/m^2^ [8]. Curiously, there is recent NAFLD literature that supports similar metabolic profiles of non-obese patients with obese patients. Some investigations posit that non-obese patients have lower levels of BMI, waist circumstance (WC), triglycerides (TG), total cholesterol (CHOL), fasting blood glucose (FBG), alanine aminotransferase (ALT), and histological indications such as steatosis, inflammation and fibrosis yet similar metabolic profile [9,10]. Of note, NAFLD prevalence appeared higher in the non-overweight females’ group vs. male group, indicating a difference with regard to biological sex, when separating overweight and non-overweight individuals in a recent study [11].

The primary goal of the present review is to evaluate cardiovascular disease risk in association with nonalcoholic fatty liver disease. This literature reviews CVD comorbidities in NAFLD patients including an analysis of hypertension, diabetes mellitus, metabolic syndrome, coronary artery disease, heart failure, and more. Systemic inflammation, endothelial dysfunction, dyslipidemia, and altered glucose metabolism act as biophysiological factors to precipitate CVD in NAFLD while genetics and heritable risk factors have been connected to NAFLD and CVD. Currently, NAFLD treatments remain absent yet the interconnection between NAFLD and CVD postulates that diabetes treatment, relevant lifestyle adjustments, and medications such as antihypertensives may prove useful. A review of CVD and NAFLD will provide a greater understanding of the interplay between disease states and aim to improve clinical management of patients, public health directives, burdens of NAFLD and CVD, and future clinical studies and literature.

## 2. NAFLD Epidemiology

Over the past few decades, the global prevalence of obesity has contributed to the rise of nonalcoholic fatty liver disease (NAFLD) as the most common liver disease worldwide and the second most common indication for liver transplant. NAFLD affects about 25% of the global population (95% confidence interval 22.10–28.65) [12,13]. The prevalence of NAFLD worldwide is 25%, with the lowest prevalence in Africa (14%), Europe (23%), and the United States (24%), with higher prevalence in South America (31%), and the Middle East (32%). In Asia, the prevalence overall is 27%; however, the prevalence of NAFLD is much higher in the urban areas of India and China than in the rural locations in those countries [13]. In the United States, the prevalence of NAFLD varies based on race: 45% Hispanic Americans, 33% Caucasian American, and 25% African Americans [14]. Researchers also found that Mexican Latinos had higher rates of NAFLD (22%) than did Puerto Rican (15.8%), Dominican (15%), Cuban (16.5%), Central American (21%), or South American (17.8%) Latinos [14]. African Americans have a lower rate of NAFLD despite having higher rates of medically co-morbid conditions associated with NAFLD, such as obesity and type 2 diabetes mellitus [15,16].

Genome-wide association studies (GWAS) have uncovered a strong association between the Patatin-like phospholipase domain containing 3 (PNPLA3) gene and NAFLD incidence [17]. A follow-up prospective cohort study showed that the PNPLA3 gene was associated with increased risk of liver-related decompensation and death [18,19]. The PNPLA3 gene did not have any effect on cardiovascular events or overall mortality [18]. People of Mexican descent have higher frequencies of the PNPLA3 gene than do non-Hispanic white or non-Hispanic black people, leading to increased risk of NAFLD incidence and progression. [20]

The prevalence of NAFLD appears to be higher in men than in women; however, when separating the data into overweight and non-overweight individuals, the non-overweight cohort had a higher prevalence of females with NAFLD [11]. Post-menopausal females have an increased risk of NAFLD because they lose their premenopausal estrogen that inhibited stellate cell activation and fibrinogenesis [21]. In a cohort of liver biopsy proven non-alcoholic steatohepatitis (NASH) patients, postmenopausal women and men had equally severe liver fibrosis [11]. Premenopausal women had less severe liver fibrosis than men [21]. In premenopausal women, elevated testosterone conferred a 2-fold higher risk of NASH and NASH fibrosis, representing an early risk factor in NASH progression in young women [22]. Premenopausal women with PCOS also carry a higher risk of NAFLD than healthy controls (34–70% vs. 14–34%) [23]. Diagnosing and preventing NAFLD in women is especially important because they have a 1.5× higher likelihood of all-cause mortality and a 2× higher likelihood of mortality from a cardiovascular event [24].

NAFLD incidence has increased dramatically over the past two decades. In a community population study based out of Minnesota starting in 1997, researchers observed a 5-fold increase in the incidence of NAFLD, with a particularly dramatic increase of NAFLD incidence in younger adults aged 18–39 [25]. The increase in NAFLD incidence among young adults is a worldwide phenomenon, with an estimated annual percentage increase of around 1% in both men and women between 1990 and 2017 [26]. As discussed later in this review, studies also show that metabolic conditions increase the incidence of NAFLD, with, for example, up to 60% of patients with diabetes found to develop NAFLD [27]. Further, an increase in patients with NAFLD and Metabolic Associated Fatty Liver Disease (MAFLD) leads to increases in cardiovascular events [28].

NASH is the inflammatory subtype of NAFLD. NASH can only be identified via liver biopsy, which shows hepatocyte ballooning and inflammation with or without fibrosis [29]. Because a NASH diagnosis requires a liver biopsy, determining the population-wide prevalence of NASH is difficult. A recent prospective cohort study of asymptomatic middle aged US adult demonstrated an NAFLD prevalence of 38% and NASH prevalence of 14% in those patients with NAFLD, a 12.2% increase from a similar study conducted ten years prior [30,31]. In a recent randomized controlled trial of simtuzumab (which was deemed ineffective), researchers monitored the long-term progression of patients with NASH. In 475 patients with NASH, progression of the disease to cirrhosis occurred in 22% of Fibrosis scale 3 patients (n = 217) and liver-related clinical events occurred in 19% of patients with cirrhosis (n = 258) over 96 weeks [32]. Models predict that the incidence of NASH will increase 7% between 2015 and 2030, leading to a 168% increased incidence of decompensated cirrhosis, 137% increased incidence of hepatocellular carcinoma, and 178% increased incidence of liver related deaths by 2030 [33].

## 3. Overall NAFLD Cardiac Complications

Cardiac complications of NAFLD have been documented, but it remains unclear whether this translates to increased cardiac mortality. A meta-analysis published in 2016 containing 34 studies and 164,949 subjects demonstrated that NAFLD was significantly associated with increased incidence of cardiovascular disease (CVD), increased incidence of hypertension, and increased prevalence of atherosclerosis [34]. However, the meta-analysis demonstrated no significant relationship between NAFLD and CVD mortality or overall mortality. In the same year, Targher et al. (2016) demonstrated the opposite with their meta-analysis, which contained 16 studies and 34,043 subjects; they found that NAFLD was associated with adverse fatal and non-fatal cardiac events and that the correlation strengthened with the severity of NAFLD [4]. More recent meta-analyses perpetuated debate about cardiac mortality, including Liu et al.’s (2019) meta-analysis, which included 14 studies and 498,501 subjects and demonstrated no significant associations between NAFLD and CVD mortality. However, this study did demonstrate elevated risk of all-cause mortality compared to those without NAFLD (HR = 1.34, 95% CI 1.17–1.54) [35]. Despite the debate about CVD mortality and NAFLD, research has explored the relationship between NAFLD and specific cardiac complications, including Atherosclerotic Cardiovascular Disease (ASCVD) risk, heart failure, arrhythmias, Hypertension (HTN), Coronary Artery Disease (CAD)/atherosclerosis, and Myocardial Infarction (MI). Table 1 summarizes overall complication.

### 3.1. General ASCVD Scores

Many cohort and cross-sectional studies have demonstrated independent associations between NAFLD and higher ASCVD scores [36,37,38]. The specific ASCVD risk score calculators and NAFLD severity measurements differed between studies. The risk calculators for ASCVD risk included American Heart Association (AHA)/American College of Cardiology (ACC) risk calculator, ASCVD risk score, the Framingham Risk Score, and the Korean Risk Prediction Model. Methods of evaluating NAFLD severity included biopsy-proven NAFLD, FIB-4 scores, other NAFLD fibrosis scores, and various forms of ultrasound graded severity. The heterogeneity of these studies furthered debate about a causal relationship, especially given that some studies used direct measures of NAFLD (biopsy) while others used indirect measures (FIB-4 scores) [39]. Recent studies have shown that certain measures of hepatic fibrosis, including the FIB-4 score, Forns index, and Hepamet fibrosis scores positively correlate with CVD risk [40]. Emerging data suggest that the effects of NAFLD on CVD risk may be misattributed and instead due to MALFD [41,42]. MAFLD is a relatively new term and encompasses hepatic steatosis and one of the following: Type 2 diabetes, overweight/obesity, or clinical evidence of metabolic dysregulation [43,44]. MAFLD and NAFLD overlap, but previous studies have argued that metabolic syndrome (MetS) or metabolic comorbidities are more important than hepatic steatosis in developing ASCVD risk.

### 3.2. Heart Failure

Three meta-analyses have demonstrated a relationship between heart failure and NAFLD. The meta-analysis by Wijarnpreecha et al. (2018) contained 12 studies and 280,645 subjects and found a relationship between NAFLD and diastolic dysfunction. Western countries had a higher odds ratio 1.76 (95% CI, 1.14–2.72) compared to subjects from eastern countries with an odds ratio of 2.59 (95% CI, 1.42–4.69), suggesting that genetic or environmental factors contribute to this association [45]. The other two meta-analyses focused on all causes of heart failure and suggested an independent relationship. The meta-analysis by Salah et al. (2022) contained 5 studies with 1,433,066 total subjects and demonstrated an association between all cause heart failure and NAFLD, with an odds ratio of 1.60 (95% CI 1.24 to 2.05) [46]. The meta-analysis by Alon et al. (2022), which contained 20 studies, found an odds ratio of 1.62 (95% CI: 1.43–1.84) [47]. All three meta-analyses demonstrated that NAFLD and heart failure are related.

### 3.3. Myocardial Infarction

The prevalence of MI is estimated to be 1.72% of the world’s population, approximately 126 million cases [48]. Many studies have argued that NAFLD increases risk for MI, including multiple cohort studies and one meta-analysis [47,49,50,51]. The recent meta-analysis by Alon et al. (2022) reported an increased risk of MI with an odds ratio of 1.66 (95% CI: 1.39–1.99) compared to those without NAFLD [47]. Emerging data suggest the severity of NAFLD may be correlated with increased MI risk, as higher FIB-4 scores have been associated with increased MI risk [50,51]. The FIB-4 score was developed to estimate the extent of liver fibrosis and includes laboratory measurements of AST, ALT, and platelet count in addition to age [52]. Data also supports that increased fatty liver index scores confer higher risk of MI in patients with NAFLD [49,53]. The fatty liver index score was created to estimate the extent of hepatic steatosis and includes measurements of BMI, waist circumference, GGT, and triglycerides [54]. These studies support that NAFLD is associated with increased incidence of MI, and this risk appears to be correlated with severity of disease.

### 3.4. Cardiac Arrhythmias

NAFLD and some arrhythmias have documented associations, especially the relationship between NAFLD and Atrial Fibrillation (AFib). AFib is the world’s most common cardiac arrythmia and is estimated to affect approximately 0.51% of people globally [55]. Associations between AFib and NAFLD have been demonstrated in four meta-analyses, the largest of which, by Gong et al. (2021), included 7,012,960 subjects [47,56,57]. The odds ratios from these meta-analyses suggest that people with NAFLD have approximately 1.2 to 2 times greater risk of developing AFib than individuals without NAFLD. AFib remains the most studied arrhythmia associated with NAFLD, but the meta-analysis by Gong et al. (2021) also demonstrated increased risk of prolonged QT intervals (odds ratio 2.86, 95% CI: 1.64–4.99), PACs/PVCs (odds ratio 2.53, 95% CI: 1.70–3.78), and heart block (odds ratio 2.65, 95% CI: 1.88–3.72) [58]. This meta-analysis was the only one we found that included arrhythmias other than AFib. NAFLD appears to be an important risk factor in the development of arrhythmias, but more research is needed to explore the risk of arrhythmias other than AFib.

### 3.5. Coronary Artery Disease

Based on epidemiologic data, NAFLD is associated with increased fatal and non-fatal cardiovascular events, including CAD (OR 1.64 95% CI 1.26–2.13) [4]. Patients with steatosis and elevated gamma-glutamyltransferase (GGT) or increasing fibrosis on liver biopsy had even higher rates of cardiovascular events (OR 2.58 95% CI 1.78–3.75) [4]. Patients with NAFLD have a higher risk of high-risk coronary artery plaques than a non-NAFLD population with similar risk factors when analyzed by CT angiography (59.3% vs. 19.0% OR 2.13 95% CI 1.18–3.85) [59]. Early detection of NAFLD after CAD detected by coronary artery calcium scans has not shown correlation thus far [60]. Genome-wide association studies (GWAS) have begun to confirm the causal relationship between NAFLD and CAD and have made strides to elucidate the relationship between NAFLD and CAD. Using the UK Biobank, researchers have discovered 94 independent GWAS loci, which suggest a causal relationship between NAFLD and CAD [61]. Studies continue to emerge in the field of GWAS to further our understanding of NAFLD and CAD.

## 4. NAFLD Pathophysiology of Cardiac Disease

**Figure 1:**
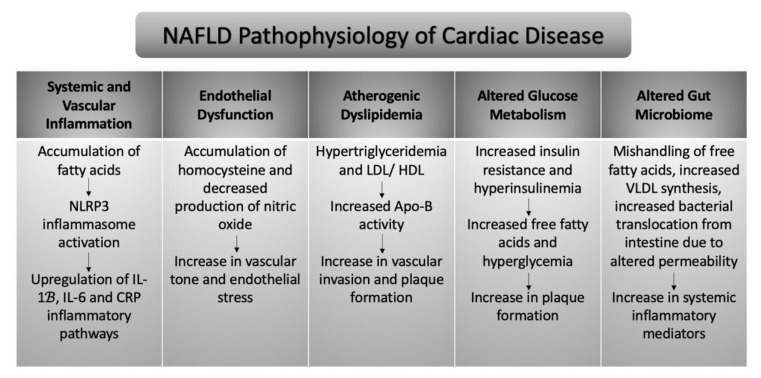
NAFLD pathophysiology of cardiac disease.

Though NAFLD is highly comorbid with other metabolic and cardiac risk factors such as obesity, Type 2 Diabetes, and a western style dietary pattern, the condition of NAFLD itself confers an increased risk for cardiac disease [62]. Many biochemical and physiological factors are at play in the relationship between NAFLD and cardiac disease. Several of the principal causes are discussed below. The overarching pathophysiology of each relationship is summarized in Figure 1.

### 4.1. Systemic and Vascular Inflammation

It is well established that systemic and vascular inflammation lead to increased plaque and clot formation and act as a main driver for the development of cardiac disease. The milieu of systemic inflammation brought on by NAFLD is a combination of a failure of the liver to properly metabolize certain vaso-altering substances or properly clear inflammatory cytokines from the blood. A principal way this inflammatory state comes about is the accumulation of fatty substances inside the liver parenchyma affecting the proper function of the liver. The inadequate uptake of lipids and altered secretion of certain fatty acids combined with insufficient fatty acid oxidation leads to systemic and vascular inflammation via the pathways described below [63]. Apolipoprotein excess has been linked to increased risk of CVD [64]. The mishandling and subsequent accumulation of apolipoprotein 3 (Apo3) acts as a damage-associated molecular pattern (DAMP) that activates TLRs 2 and 4 by dimerization, thus activating the NLRP3 inflammasome [64]. In its usual role, the NLRP3 inflammasome modulates the activity of caspase-1, an IL-1Beta converting enzyme, activating pro-inflammatory cytokines. In the setting of NAFLD, the increased Apo3 over-activates the NLRP3 inflammasome, increasing flux through the IL-1 to IL-6 to CRP pathway [64]. Other fatty acids, such as palmitic acid, can also activate the NLRP3 inflammasome, representing another distinct pathway for production of inflammatory cytokines [65]. Studies have shown that higher concentrations of palmitic acid are linked to higher rates of cardiovascular mortality, likely due to the increased activity of inflammation through the pathway described above [66]. The mishandling and accumulation of fatty acids in NAFLD lead to higher levels of systemic inflammatory mediators, increasing vascular inflammation and tone, the development of atherosclerotic plaques, and the risk for CVD.

### 4.2. Endothelial Dysfunction

Endothelial dysfunction is one of the first steps in developing atherosclerosis. In patients with NAFLD, increased endothelial dysfunction is a sequela of the liver’s inability to regulate vaso-inflammatory substances or produce protective substances like nitric oxide. The liver in its normal state is able to conjugate Methionine s-adenosylmethionine (SAM), which is a common source of methyl in other metabolic reactions, such as the synthesis of phosphatidylcholine and creatine. When SAM is subsequently demethylated, it forms s-adenosylhomocysteine (SAH) and homocysteine. Though the exact mechanism is unknown, it is believed that the above pathways of metabolizing Methionine are altered in patients with NAFLD, leading to high levels of homocysteine in many patients [67]. Meta-analyses have shown that NAFLD patients have consistently higher levels of homocysteine compared to controls [68]. Once homocysteine begins to accumulate in the bloodstream, several deleterious effects on coagulation and inflammation occur. High levels of homocysteine in the blood have been shown to cause oxidative damage to platelets by decreasing the available stores of glutathione, thus causing an increase in platelet activation and a hypercoagulable state [69]. Homocysteine has also been associated with increased endothelial cell dysfunction, impaired protein folding, and increased oxidative stress, providing another avenue for ongoing inflammation. Hyperhomocysteinemia has deleterious effects even on the liver, increasing intrahepatic vascular tone and inhibiting the production of nitric oxide, leading to even further widespread vascular stress [70].

NAFLD also causes endothelial dysfunction via decreased levels of the vaso-protective substance nitric oxide due to the liver’s inability to break down certain metabolites. Nitric oxide acts locally as a vasodilator by activating guanylate cyclase, cleaving a phosphate group from guanosine triphosphate (GTP) to be used for phosphorylation and relaxation of vascular smooth muscle. However, patients with NAFLD have increased levels of asymmetric dimethyl arginine (ADMA), which is a natural antagonist to nitric oxide synthase. These increased levels of ADMA are due to impaired hepatic breakdown of the substance, leading to over-inhibition of nitric oxide synthase and a subsequent increase in vascular stress and damage [71]. Endothelial dysfunction is an important step in forming atherosclerotic plaques, and in NAFLD, this step is greatly accelerated by hyperhomocysteinemia and decreased levels of nitric oxide.

### 4.3. Atherogenic Dyslipidemia

Hepatic lipid metabolism changes that lead to NAFLD also result in atherogenic dyslipidemia, characterized by plasma hypertriglyceridemia, increased small dense low-density lipoprotein (LDL) particles, and decreased high-density lipoprotein cholesterol (HDL-C) [72,73]. In NAFLD, increased hepatic triglycerides are due to insulin resistance, which leads to increased peripheral lipolysis and den novo lipogenesis (DNL). Elevated plasma insulin and glucose drive DNL via Liver X-Receptor (LXR) and carbohydrate response element binding protein (ChREBP) pathways [74]. DNL increases malonyl-coA via ACC. Malonyl-coA then inhibits CPT1, decreasing fatty acid oxidation and mitochondrial function [75]. Fatty acid availability is further increased by lipoprotein lipase (LPL) inhibition through overexpression of ANGPTL8 and 3, also produced through the LXR pathway. ANGPTL8 also decreases triglyceride hydrolysis via adipose triglyceride lipase These processes increase hepatic triglycerides, thereby increasing hepatic triglyceride secretion and plasma triglyceride level [76].

In adipocytes, triglycerides are hydrolyzed by lipases into free fatty acids (FFA). FFA enter the bloodstream and subsequently enter the liver and muscles [77]. Upon re-esterification of FFA with posttranslational stabilization of ApoB, very low-density lipoprotein (VLDL) particles form. VLDL production is further amplified by hyperglycemia [78]. Specifically, the liver produces larger triglyceride-rich VLDL-1 and smaller triglyceride-poor VLDL-1. Atherogenic dyslidemia, insulin resistance, and type 2 diabetes are characterized by overproduction of VLDL-1. VLDL-1 overproduction is a central driver in atherosclerosis, as it changes levels of other lipoproteins, including increased LDL and decreased HDL [78,79]. Increased hepatic cholesterol, namely intracellular VLDL-1, decreases LDLR and PCSK9 mRNA expression via inhibition of the SREBP2 pathway. Posttranscriptional regulation of PSCK9 also decreases membrane bound LDLR. Downregulation of LDL receptors decreases intracellular uptake of LDL with a consequent rise in plasma levels [80,81].

Elevated plasma triglycerides, remnant lipoprotein cholesterol levels, and small dense LDL particles all invade the arterial wall and cause atherosclerotic plaques. Apo-B containing lipoproteins, most notably VLDL1 and LDL, act as damage-associated molecular patterns (DAMPs) to activated Toll-like receptors (TLRs), resulting in a cascade of inflammatory pathways [64]. Additionally, triglyceride-rich lipoproteins containing apolipoprotein C3 (ApoC3) activate TLRs 2 and 4 via dimerization. TLRs 2 and 4, DAMPs, pathogen associated molecular patterns (PAMPs), activate the NOD-like receptor family and pyrin domain-containing protein 3 (NLRP3) inflammasome, which regulate the activity of the enzyme caspase-1, also known as interleukin (IL)-1β converting enzyme [64,82]. Caspase-1, via proteolytic activation, increases the production of proinflammatory cytokines through the IL-1, IL-6, and CRP pathways, leading to endothelial cell damage and atherosclerotic disease [83]. Therefore, upregulation of TLRs by an atherogenic favoring lipid profile, and the resultant production of inflammatory cytokines via the NLRP3 inflammasome, is an important link between atherogenic dyslipidemia and the development of CVD in NAFLD patients [83]. Plaque formation is further exacerbated by oxidized LDL, which is derived from LDL or small dense LDL (sdLDL). Oxidized LDL interacts with scavenger receptors, macrophages, and smooth muscle cells, causing a large deposition of cholesterol within the blood vessel [84]. These plaques then undergo lipid-deposition, inflammation, fibrosis, and calcification over decades [85]. Another mechanism leading to upregulation of TLRs 2 and 4 in NAFLD is seen via increased hepatic DNL. Notably, hepatic DNL in NAFLD leads to increased saturated fatty acid, specifically palmitic acid (C 16:0), flux, and density in VLDL particles [64,65]. Saturated fatty acids trigger further endothelial inflammation via TLRs 2 and 4, contributing to vascular injury and atherosclerosis in NAFLD [64,86]. Notably, higher concentrations of palmitic acid or palmitoleic acid (16:1n-7) correlated positively with all cause and cardiovascular mortality in a stepwise fashion [66,87,88].

### 4.4. Altered Glucose Metabolism

Hallmarks in the pathogenesis of NAFLD are altered glucose metabolism and hepatic insulin resistance [70,71]. The mechanisms of insulin resistance in NAFLD have been well studied and can be attributed to systemic inflammation, visceral obesity, increased body weight, and maladaptive ectopic fatty tissue [64,89]. Ectopic fat not only accumulates in the liver but also in the pancreas, leading to insulin resistance and beta cell dysfunction [89,90]. Additionally, hyperinsulinemia ensues due to insulin resistance, contributing to the development of CVD [64]. Specifically, hyperinsulinemia drives hepatic glucose production, raising plasma levels of glucose, which then stimulates more insulin production. This self-reinforcing cycle is exacerbated by a decreased clearance of insulin by the liver in NAFLD. Additionally, insulin activates transcription factors sterol regulatory element-binding protein 1c (SREBP-1c) and carbohydrate-responsive element binding protein (ChREBP), which increase flux of DNL via lipogenic enzyme production [91]. The combination of hepatic fat accumulation and increased saturated fatty acids, in addition to persistent hyperglycemia, is a central driver in the altered metabolic profile of NAFLD. This altered metabolic profile, as detailed earlier, is central to the development of CVD.

As it relates directly to plaque formation, insulin resistance results in oxidative stress and severe inflammation through multiple pathways. Upregulation of inflammatory signaling pathways, inflammasome activation, and increased production of advanced glycosylation end products (AEG) are all consequences of persistent hyperglycemia and postprandial glucose spikes [64,90]. Additionally, insulin resistance has been associated with cardiac autonomic neuropathy due to renin-angiotensin-aldosterone system dysregulation and fibrinolytic dysfunction. This may favor a microenvironment that results in systolic and diastolic dysfunction, cardiac arrhythmias, and endothelial dysfunction [64,92,93,94].

### 4.5. Plaque Formation in the Setting of an Altered Gut Microbiome

A recent study investigated how altered gut microbiomes in patients with NAFLD affect normal free cholesterol metabolism [95]. High levels of triglycerides are associated with both calcified and non-calcified plaque formation [96]. Part of the reason patients with NAFLD have a high level of triglycerides is that their altered gut microbiome increases choline metabolism, and as choline is essential to VLDL synthesis, this lack of VLDL secretion limits triglyceride hydrolyzation and clearance [95]. Dongiovanni et al. have theorized that VLDL synthesis is impaired due in large part to lipid oxidative DNA damage [96]. Another connection between CVD and NAFLD is shown in the deranged metabolism of choline and carnitine as represented by trimethylamine oxide (TMAO) levels within the gut microbiome. TMAO is typically a marker of severity and risk for cardiovascular events. There is need for further research in this is an area, but it has already been shown to be positively correlated with the severity of disease in NAFLD [96]. In addition to TMAOs, short-chain fatty acids and bile acids can have disrupted metabolism and signaling. This may lead to an inability to properly metabolize components of red meat, leading to a higher risk of atherosclerosis [97]. Additionally, there is some evidence that higher levels of ApoC3 in patients with NAFLD may lead to increased activation of inflammatory pathways, further contributing to plaque formation [96].

The increased production of ethanol in patients with NAFLD leads to intestinal tissue permeability, which allows PAMPs derived from the gut to enter portal circulation and descend on the liver [95]. PAMPs damage and fibrose the liver by inducing their standard inflammatory pathway, leading to increased levels of TNF-alpha, interleukin (IL-6), monocyte chemotactic protein-1, and C-reaction protein. These proinflammatory markers are linked to endothelial dysfunction and lead to vascular plaque formation [95]. Any environment that leads to systemic inflammation can lead to clotting and plaque formation by endothelial dysfunction and altered vascular tone [96]. Further, metalloproteinases and homocysteine, which are also pro-atherosclerotic, are increased in patients with NAFLD [95]. In addition to leading to an increased risk of atherosclerotic plaque formation, NAFLD is associated with worse clinical outcomes in the event that the plaque ruptures and leads to an acute coronary event [97].

### 4.6. Genetic Susceptibility

Originally, Tarnoki et al. studied 208 adult Hungarian twins who were monozygotic or dizygotic, and heritability analysis found no genetic component to ultrasound diagnosed NAFLD [98]. Later, a study of overweight children with biopsy proven NAFLD and overweight children without NAFLD found that when adjusted for age, sex, race, and BMI, the heritability of fatty liver was 1.000 [99]. The heritability of hepatic steatosis was found to be 0.52 in a study of 60 pairs of community-dwelling twins. In a meta-analysis of data, CT-diagnosed hepatic steatosis was found to have a heritability of 0.26–0.27 [100]. In one study utilizing data from the Framingham Heart Study, participants with at least one parent with hepatic steatosis had nearly two-fold increased odds of hepatic steatosis compared to participants without a parental history of hepatic steatosis [101]. Studies have also measured the heritability of NAFLD/hepatic steatosis in African Americans and Hispanic Americans. Wagenknecht et al. found that CT-scan diagnosed NAFLD was modestly heritable, with 0.21 in Hispanic Americans (n = 795) and 0.19 in African Americans (n = 347) [102]. Another study in 2013 found that CT-measured hepatic steatosis was 0.22–0.34 heritable in three African American cohorts (n = 836, 965, 405) and 0.20 in one Hispanic American cohort (n = 849) [103].

Multiple studies have also established the heritability of risk factors associated with NAFLD. One twin study found that blood pressure, triglycerides, glucose, insulin resistance, hemoglobin A1C, and high-density lipids had significant shared gene effects with hepatic steatosis (n = 130) [104]. Makkonen et al. found that in a study of 313 individual twins, the heritability of S-ALT was 0.55 and fS-insulin was 0.61. In 66 of these subjects, liver fat was measured, and S-ALT and fS-insulin were correlated with liver fat content [105]. Another twin study of Danish twins found that ALT, LDH, GGT, and bilirubin are 0.35–0.61 heritable (n = 580) and that adjustment for alcohol consumption and BMI had no influence on heritability [106]. In a study of 157 familial combined hyperlipidemia and 20 spouses, heritability calculations revealed 0.20–0.36 of the variability in ALT levels was attributed to genetic factors [107].

The frequency of NAFLD varies significantly between ethnicities, with the general pattern being Hispanic Americans > White Americans > African Americans. Browning et al. examined magnetic resonance spectroscopy-identified hepatic steatosis among 2287 multiethnic subjects and showed that 45% of Hispanic Americans, 33% of White Americans, and 24% of African Americans had hepatic steatosis [108]. A study of 795 Hispanic Americans and 347 African Americans found that NAFLD was more common in Hispanic Americans (24%) and African Americans (10%) [102]. Fleishman et al. compared the prevalence rates of NAFLD between Hispanics of Mexican origin (33%) and Hispanics of Dominican (16%) and Puerto Rican (18%) origin in 788 participants. Even after controlling for age, sex, BMI, waist circumference, hypertension, serum HDL, triglyceride, CRP level, and insulin resistance, Mexican Americans remained significantly more likely to have NAFLD than Dominicans and Puerto Ricans [109].

A genome-wide association scan of nonsynonymous sequence variations in a multiethnic population (n = 9229) found that an allele in PNPLA3 was strongly associated with increased hepatic fat levels and hepatic inflammation. This study found that the allele was most common in Hispanic Americans. A different allele was associated with lower hepatic fat content in African Americans [110]. In a very diverse study of 4804 adults, it was shown that there is and NCAN and ultrasound-measured hepatic steatosis. Variants in PNPLA3, NCAN, GCKR, and PPP1R3B were associated with non-Hispanic whites. PNPLA3 variants were associated with Mexican Americans [111]. Palmer et al. found an association between variants in or near PNPLA3, NCAN, GCKR, and PPP1R3B and CT-measured hepatic steatosis in African Americans and an association between variants in or near PNPLA3 and PPP1R3B and CT-measured hepatic steatosis in Hispanic Americans [103]. A variant in the TM6SF2 gene was associated with high hepatic fat content diagnosed by MRI in White Americans and African American obese children (n = 454). A joint effect was observed among TM6SF2, PNPLA3, and GCKR determining intrahepatic fat accumulation [112]. A meta-analysis of 9 papers found lower risks of NAFLD for patients with the CC genotype of the TM6SF2 gene, a transmembrane 6 superfamily 2 gene, which had previously been linked with lipid metabolism [113].

Certain genetic variants are also associated with more aggressive NAFLD. A meta-analysis of 16 studies with 2937 subjects found that variations in PNPLA3 exerted a strong influence on liver fat accumulation and on susceptibility of more aggressive disease [114]. A study of 502 European NAFLD patients found that carriage of SDO2 C47T polymorphism was associated with more advanced fibrosis in NASH. PNPLA3 was also associated with susceptibility to advanced fibrotic disease [115]. A study of 702 patients with biopsy proven NAFLD and 310 healthy controls from Italy and the UK found that the Lys121Gln ENPP1/PC1 and IRS-1 972Arg polymorphisms affecting insulin receptor activity predisposed patients with diabetes to liver damage and decreased hepatic insulin [116]. Miele et al. found that in a study of 415 patients, KLF6 expression increased with increased steatosis, inflammation, and fibrosis in NAFLD livers [117].

## 5. Cardiovascular Comorbidities in NAFLD Patients and Preventive Strategies to Lower ASCVD

### 5.1. Hypertension

Hypertension is estimated to affect more than 31% of adults across the world and is the most common risk factor for CVD [118,119]. Recent evidence suggests a significant association between NAFLD and hypertension. An observational study estimated the prevalence of NAFLD to be nearly 50% among patients with hypertension [120]. Both in this patient cohort and in a larger population study, hypertension was significantly more prevalent among patients with NAFLD than in patients without NAFLD [121]. Additional cross-sectional studies have demonstrated a similar association between NAFLD and increased blood pressure among normotensive individuals with elevated blood pressure and pre-hypertensive individuals [122,123]. A 2022 meta-analysis of more than 390,000 patients indicated that NAFLD is associated with an approximately 1.6-fold increased risk for developing hypertension [124]. While the association between NAFLD and arterial hypertension is well supported by clinical evidence, the precise relationship in terms of causality between the two remains unclear.

The 2017 American College of Cardiology/American Heart Association High Blood Pressure Guidelines do not list NAFLD as a cause of hypertension [125]. However, emerging evidence suggests that preexisting NAFLD may contribute to the development of hypertension [126]. In a large prospective analysis of more than 22,000 non-hypertensive males with varying degrees of baseline NAFLD diagnosed by ultrasound, patients with NAFLD were more likely to develop hypertension after 5 years of follow-up than patients without NAFLD [127]. This association was more significant in patients with moderate to severe NAFLD compared to mild NAFLD (hazard ratio, 1.14 vs. 1.07), suggesting that the development of hypertension might be more strongly associated with more severe NAFLD. A large retrospective cohort analysis found similar results after 5 years of follow-up, demonstrating that NAFLD was associated with the development of incident hypertension (odds ratio, 1.36) [128]. Resolution of fatty liver at follow-up was not associated with increased incidence of hypertension, further supporting that NAFLD is likely an independent risk factor for developing hypertension. Additionally, NAFLD has been found to independently increase the risk of developing elevated systolic blood pressure among normotensive individuals (odds ratio, 2.13) [120]. The pathophysiology of NAFLD as a causative contributor to incident hypertension is still a topic of investigation, but preliminary studies suggest etiological factors may include NAFLD-induced systemic inflammation, insulin resistance, oxidative stress, increased vasoconstriction, decreased vasodilation, and arterial stiffness [126,129,130,131,132].

Conversely, multiple studies have demonstrated that hypertension may contribute to the development of NAFLD. Two large prospective cohort studies have found hypertension to be an independent predictor of incident NAFLD (relative risk and hazard ratio, 1.31 and 1.75, respectively) [133,134]. Another small prospective cohort study showed hypertension to be an independent risk factor for the progression of liver fibrosis (odds ratio, 4.8) [135]. In a retrospective study of patients with NAFLD diagnosed using transient elastography, the prevalence NAFLD was progressively increased among higher tiers of blood pressure, suggesting a correlation between blood pressure severity and risk of developing NAFLD [136]. Further evidence suggests that NAFLD and hypertension might be independent risk factors for each other. A prospective study involving a subgroup for participants from the Framingham Heart Study demonstrated a bidirectional relationship between NAFLD and hypertension after a follow up period of six years [137]. Specifically, NAFLD was identified as a risk factor for developing hypertension (odds ratio, 1.42), while hypertension was shown in a parallel analysis to be a risk factor for incident NAFLD (odds ratio, 3.34). Two additional studies demonstrated similar bidirectional relationships between NAFLD and hypertension [138,139]. This is due to a cyclical progression of disease, such that increasing blood pressure status facilitates liver damage and vice versa. Additional prospective investigation is needed to further elucidate the precise relationship between these two diseases.

Evidence-based approaches for managing coexistent NAFLD and hypertension remain limited, and there are no current recommendation guidelines for pharmacologic or nonpharmacological treatment for these individuals. Lifestyle adjustments, including nutritional changes, improved physical activity, and weight loss continue to be the cornerstone of NAFLD therapy [140,141]. Numerous trials of specialized dietary programs including the Dietary Approaches to Stop Hypertension (DASH) Diet and the Mediterranean Dietary Pattern (MedDiet) have shown success in reducing hypertension risk, thereby reducing the risk of developing hepatic steatosis NAFLD and improving other cardiovascular comorbidities among individuals with NAFLD [142,143,144,145]. However, prospective investigation into the efficacy of the DASH diet for managing NAFLD is limited. One randomized controlled trial demonstrated that overweight/obese subjects with NAFLD in a DASH diet group had higher degrees of improvements in liver enzymes, insulin sensitivity, and body weight compared to matched subjects in a low-energy diet group [146]. Unfortunately, this study did not evaluate outcomes of specific histologic or radiographic features of NAFLD. The MedDiet has been found to reduce the degree of hepatic steatosis among patients with NAFLD in a crossover trial [147]. However, this study only evaluated a dietary period of six weeks, and the reductions in hepatic fat content were only significant up until the 6-month follow-up. Other studies with follow-up periods up to 6 years have suggested that the MedDiet may be associated with reduced prevalence of NAFLD and decreased degree of hepatic steatosis [148,149].

Regarding pharmacologic therapy for coexistent NAFLD and hypertension, renin-angiotensin-aldosterone system (RAAS) inhibitors have shown some favorability. This is predicated on the current understanding that upregulation of the RAA system seems to be involved in the pathogenesis of NAFLD [150]. One small study of seven patients with coexistent NASH and hypertension treated for 48 weeks with losartan demonstrated improved blood markers of hepatic fibrosis and improved hepatic necroinflammation and fibrosis as determined by biopsy [151]. Larger studies have shown similar favorable results in improving hepatic fibrosis, liver stiffness, and other markers of NAFLD after treatment with either angiotensin-converting enzyme (ACE) inhibitors or angiotensin receptor blockers (ARBs) [152,153,154].

Several newer biologic therapies have been evaluated in phase II and phase III clinical trials [155]. Modulators of the farnesoid X receptor (FXR) are thought to be involved in the pathogenesis of hepatic fibrosis and are the topic of several clinical trials. Obeticholic acid is an FXR agonist currently under investigation in two Phase 3 clinical trials to evaluate the efficacy in improving hepatic fibrosis [156,157]. A previous trial has demonstrated that obeticholic acid improved systolic blood pressure and reduced the degree of necroinflammation in patients with NASH [158]. Analogs of fibroblast growth factor 19/21 (FGF 19/21) are also currently undergoing phase II clinical trials to evaluate utility in improving fibrosis in NASH patients [159,160]. FGF21 is thought to reduce progression of NAFLD, and positive results have already been demonstrated in a small phase II trial of the PEGylated FGF21 analog Pegbelfermin [161]. Additional agents of interest in managing hepatic fibrosis were PPARα/δ agonist Elafibranor and the CCR2/5 antagonist cenicriviroc. Unfortunately, neither drug’s clinical trial results warranted continuing the study, and both were terminated early. Cenicriviroc was shown in a randomized placebo-controlled trial to improve fibrosis without progressing to steatohepatitis, and a preclinical study has demonstrated that a different experimental CCR2 inhibitor improved elevated blood pressure in mice [162,163]. Further preclinical and clinical investigations are ongoing and required to evaluate the efficacy of these and other biologic agents for their utility in managing coexistent NAFLD and hypertension.

### 5.2. Diabetes Mellitus

The global prevalence of type 2 diabetes mellitus (T2DM) has continued to rise and is projected to increase to 7079 individuals per 100,000 worldwide by the year 2030 [164]. The association between NAFLD and T2DM is well established. A 2015 meta-analysis of 729 studies including more than 8.5 million individuals demonstrated that the global prevalence of T2DM was 22.5% among patients with radiographically diagnosed NAFLD and greater than 43.6% among NAFLD diagnosed via biopsy [2]. A 2019 meta-analysis revealed that the prevalence of biopsy-proven NAFLD in patients with T2DM is greater than 55%, more than 2-fold higher than the general population [165]. Among the major components of metabolic syndrome, including obesity, hypertension, and hyperlipidemia, T2DM appears to be the most important risk factor for the predicting NAFLD-associated sequela, such as hepatic fibrosis [166,167]. Furthermore, T2DM has also been demonstrated to increase the risk of hepatocellular carcinoma, liver-related mortality, and overall mortality among NAFLD patients [165,168,169,170].

Multiple studies have demonstrated that the presence of NAFLD in individuals with T2DM increases the risk of CVD, independent of other components of metabolic syndrome. Among these, a prospective case–control study demonstrated a significantly higher risk for developing CVD (including MI, coronary revascularization procedures, ischemic stroke, or cardiovascular-related death) among T2DM individuals with ultrasound diagnosed NAFLD compared to those without evidence of NAFLD (odds ratio, 1.84) [171]. A larger cross-sectional study of nearly 3000 T2DM individuals conducted by the same researchers demonstrated an increased prevalence of coronary (26.6 vs. 18.3%), cerebral (20.0 vs. 13.3%), and peripheral (15.4 vs. 10.0%) vascular disease among NAFLD patients compared to those without NAFLD [172]. More recently, among a Turkish cohort of individuals with NAFLD, patients with T2DM compared to non-diabetic patients were shown to have higher mean carotid intima-media thickness, the study’s endpoint for assessment of cardiovascular risk [173]. Additionally, NAFLD was shown to be associated with an increase in the development of atrial fibrillation in patients with T2DM [174]. The results of these studies were significant after adjusting for other CVD risk factors, demonstrating that coexistent T2DM and NAFLD are independently associated with multiple subtypes of CVD.

Numerous studies have demonstrated that NAFLD increases the risk of developing T2DM, with some estimating the risk is increased approximately 2-fold to 3-fold compared to the general population [175,176,177]. One large 2010 prospective study with a mean follow-up of 11.5 years found that unadjusted NAFLD significantly increased the risk of incident T2DM (19% vs. 6%), though this became statistically nonsignificant after adjusting for confounders [178]. Multiple meta-analyses performed within the past decade have demonstrated an increased NAFLD-associated risk for developing T2DM after adjusting for confounding variables. Among these, two meta-analyses demonstrated ultrasonographic-diagnosed NAFLD increased the risk for incident T2DM (odds ratio, 3.51 and 1.86, respectively) [179,180]. A larger 2018 meta-analysis of nearly 300,000 individuals from 19 studies indicated that individuals with NAFLD have a greater than 2-fold increased risk for developing incident T2DM [181]. Within this cohort of studies, individuals with more severe ultrasonographic steatosis and greater degree of fibrosis tended to have a higher risk of developing T2DM than individuals with less severe liver disease [182,183].

At present, there is far less conclusive evidence indicating whether T2DM is a significant contributor to the development of NAFLD. In a cross-sectional analysis of nondiabetic Korean individuals, ultrasound diagnosed NAFLD was associated with increased HbA_1c_ level and insulin resistance, independent of obesity and other metabolic confounders [184]. These authors concluded that abnormal glucose tolerance and the phenomenon of pre-diabetes might contribute to the development or progression of steatosis in patients who do not meet criteria for a formal diagnosis of diabetes. Evidence has also suggested that the percentage of liver fat is higher in patients with T2DM compared to patients without T2DM, supporting the authors’ theories [185]. One recent study showed that among overweight and obese patients with NAFLD, T2DM significantly increased the risk of steatosis (48.3% vs. 17.4% and 79.9% vs. 57.6%, respectively) and moderate-to-high risk fibrosis (31.8% vs. 20.1%) [186]. However, this study exclusively evaluated overweight and obese patients and did not include patients of normal BMI. Currently, no prospective studies among the general population have demonstrated a causal relationship between T2DM and the risk for developing NAFLD. Some evidence has suggested that the risk of NAFLD might be lowered in patients with Type 1 Diabetes Mellitus (T1DM), though these reports are purely observational and are limited by differences in lipid profiles, liver enzymes, and degree of visceral adipose in study versus control groups [187,188,189].

Despite the high coexistence of NAFLD and T2DM and increased risk of cardiovascular sequela among these individuals, evidence does not currently support screening for NASH or NAFLD in patients with T2DM. Due to the lack of quality evidence-based therapeutic options and inconsistent efficacy of diagnostic modalities, a recent analysis demonstrated that screening for NAFLD in these patients is not currently cost effective [141,190]. In its 2017 practice guideline, the American Association for the Study of Liver Diseases did not recommend routine NAFLD screening for high-risk patients in primary care, diabetes, or obesity clinics [141]. No pharmacological agents are currently approved for treating coexistent NAFLD and T2DM, though utility of pharmacologic and non-pharmacologic therapies is under investigation. Given the current understanding of the pathophysiological interplay between insulin resistance in both T2DM and NAFLD, most therapeutic strategies tend to target insulin resistance. At present, lifestyle modification appears to be the most effective strategy in reducing adverse cardiovascular outcomes among individuals with coexistent NAFLD and T2DM [191]. Among T2DM patients with NAFLD, one study demonstrated that a 15% decrease in BMI through aerobic exercise and diet effectively improved liver function, marked by a reduction in liver enzymes [192]. Other researchers showed that aerobic exercise alone without change in body weight also improved liver enzymes in patients with concomitant T2DM and NAFLD and that intense exercise is more effective than moderate exercise [141,193]. However, a major limitation of lifestyle modifications is the poor adherence to exercise and weight loss, particularly among overweight and obese individuals, in whom the risk of NAFLD is greatest [194].

While evidence supporting the use of antihyperglycemic agents remains limited, multiple classes of agents have shown promise [195]. Several studies showed metformin improved liver enzymes, reduced histologic liver damage, and improved weight loss in T2DM patients with NAFLD [196,197], though a small randomized controlled trial between metformin and a placebo showed no significant improvement in liver enzymes or histopathology [198]. The experimental mitochondrial pyruvate carrier agent MSD-0602K has shown similar efficacy in reducing NAFLD histopathology after 12 months, though additional trials evaluating its longer-term efficacy in managing NAFLD and NASH are ongoing [199]. Incretin modulators, including agents from both the glucagon-like peptide-1 (GLP-1) agonist and dipeptidyl peptidase-4 (DPP-4) inhibitor classes, have recently shown promise in potentially reducing the progression of NAFLD. To date, the GLP-1 agonist liraglutide has been most widely investigated for this purpose. In a small prospective study of obese individuals with known T2DM on stable doses of metformin and evidence of hepatic steatosis, those treated for 6 months with either liraglutide or the GLP-1 agonist exenatide demonstrated reduced hepatic steatosis and decreased liver enzyme levels [200]. Studies have reported similar effectiveness of exenatide, liraglutide, and the DPP-4 inhibitor sitagliptin

### 5.3. Metabolic Syndrome

The metabolic syndrome (MetS) is characterized by a cluster of metabolic derangements including elevated blood pressure, atherogenic dyslipidemia, impaired glucose tolerance and insulin resistance, and abdominal obesity [201]. Multiple sets of varying criteria are commonly used to diagnose MetS, including those described by the World Health Organization (WHO), the American Association of Clinical Endocrinologists, the National Cholesterol Education Program’s Adult Treatment Panel III report (ATP III), and the International Diabetes Foundation [44,202,203]. The association of MetS with an increased risk of cardiovascular-associated morbidity and mortality is well established [204,205,206]. The prevalence of MetS varies based on which organizational criteria is used for diagnosis, but prevalence rates of the non-obese international population range from 26% to more than 35% [207,208]. Among obese individuals, prevalence has been estimated to be as high as 65% in women and 78% in men, with hypertension being the most frequently occurring factor contributing to MetS prevalence [209].

NAFLD is widely considered the hepatic manifestation of MetS given the large degree of metabolic overlap [210]. In one study, 88% of individuals with NAFLD had at least one component of MetS, and approximately one third had three or more MetS components [211]. Furthermore, MetS is considerably associated with the prevalence and severity of NAFLD. In a large cohort study of nearly 12,000 individuals, the prevalence of NAFLD diagnosed with ultrasound and the NAFLD Fibrosis score (NFS) was 43% greater in those with MetS (odds ratio, 11.5) [212]. These investigators also showed that the prevalence of NAFLD increased with the number of MetS components, such that the prevalence of NAFLD among individuals with all five MetS components was 67% greater than that of the general population. Additionally, the presence of MetS has been shown to increase morbidity and mortality risk in NAFLD patients. Among a cohort of NAFLD patients from the National Health and Nutrition Examination Survey (NHANES III), the presence of coexistent MetS was found to be an independent predictor of all-cause, liver-specific, and cardiovascular mortality, whereas NAFLD without MetS conferred no increased risk [213]. In another cross-sectional study, the presence of at least one MetS component increased the risk of mortality of NAFLD patients compared to individuals without MetS after both 8 and 16 years of follow-up (4.7% vs. 2.6% and 11.9% vs. 6%, respectively) [214].

Due to the multifactorial and multicomponent nature of MetS, precisely evaluating the causal relationship between MetS and NAFLD is challenging. Nevertheless, numerous studies have demonstrated that MetS and its individual components independently contribute to the development and progression of NAFLD [135,215]. The number of metabolic abnormalities also seems to be positively associated with NAFLD risk. Adjusted for lifestyle risk factors, one study associated NAFLD with either one, two, or three components of MetS (hazard ratio, 1.92, 2.64, 3.51, respectively) compared to individuals without MetS [135]. Among these metabolic abnormalities, obesity and hyperlipidemia were more highly associated with NAFLD compared to hypertension or hyperglycemia. In a 2020 study, Kim et al. demonstrated that MetS, regardless of obesity, was associated with increased progression of fibrosis among patients with NAFLD [215]. This study also showed that among individuals with biopsy proven NAFLD, non-obese metabolically abnormal individuals had similar degrees of histological abnormalities compared to obese otherwise metabolically normal counterparts. These results suggest that the diagnosis of MetS without a diagnosis of obesity contributes to clinically significant NAFLD and progression to NASH and cirrhosis.

Unfortunately, the diagnosis of NAFLD remains challenging. While the gold standard for diagnosing NAFLD is liver biopsy, most studies evaluating and risk stratifying NAFLD employ noninvasive techniques, including ultrasound, magnetic resonance elastography, or laboratory-based diagnostics. The FIB-4, NFS, and other scoring systems such as the AST/platelet ratio index (APRI) have also been used to risk stratify for liver-related morbidity in NAFLD patients [140,216]. This can complicate evaluating the causal relationship between NAFLD and MetS, particularly when NAFLD progression is assessed using one of these scoring systems, given that both NAFLD and MetS can independently elevate liver enzymes.

Evidence suggests that the relationship between MetS and NAFLD is likely bidirectional, such that preexisting NAFLD can contribute to the development and worsening of metabolic derangements and MetS [217]. Among a cohort of nearly 18,000 Chinese individuals without baseline MetS, researchers found that after 6 years of follow-up, NAFLD was an independent risk factor for the development of MetS (hazard ratio, 1.55) [218]. Additional studies have also found that NAFLD is an independent risk factor for MetS [219,220,221].

Lifestyle modification remains the primary management strategy for reducing cardiovascular risk among individuals with MetS and NAFLD. Weight loss, either due to diet alone or diet combined with physical activity, has been shown to decrease progression of hepatic steatosis, inflammation, and fibrosis [222]. Additional studies have shown that physical exercise alone without weight loss can decrease liver enzymes and steatosis [223]. Among patients with NAFLD, these lifestyle modifications have considerably decreased the risk of cardiovascular and cardiac complications [224]. Managing individual components of MetS continues to be the mainstay of reducing cardiovascular risk in NAFLD individuals. Maintaining adequate blood pressure and blood glucose control is important in mitigating cardiovascular risk in NAFLD patients and was discussed previously in this review. In addition to managing hypertension and improving overall cardiovascular risk, some evidence suggests that ARBs may improve serum liver enzyme levels and liver histology in NAFLD patients [225]. Multiple analyses have shown support for the use of anti-lipid therapies, including statins, in reducing adverse cardiovascular outcomes among NAFLD patients [226,227,228]. While fibrates have not been shown to improve histology in NAFLD patients, they have been demonstrated to decrease overall cardiovascular morbidity and mortality and are effective in managing dyslipidemia in NAFLD patients with MetS [229].

A 2016 non-systematic review suggested that bariatric surgery might be helpful in managing NAFLD in patients with MetS [230]. Multiple studies included in this review showed that bariatric surgery improved multiple metabolic comorbidities and resulted in decreased lobular inflammation, hepatic ballooning, and steatosis. Similar results have been demonstrated in small prospective studies [231]. However, these studies secondarily evaluated NAFLD and MetS outcomes in patients who otherwise had indications for bariatric surgery. A 2019 meta-analysis of 32 studies reported that bariatric surgery decreased ballooning degeneration, fibrosis, and inflammation and showed biopsy-confirmed resolution of steatosis in 66% of patients [232]. While bariatric surgery is not currently recommended as a means of managing coexistent MetS and NAFLD, further investigation with larger prospective clinical trials may show utility in select populations.

### 5.4. Coronary Artery Disease

Despite NAFLD being a liver pathology, the most common cause of death among NAFLD patients stems from CVD, mainly ischemic heart disease [233,234,235]. Previous literature has shown the prevalence of coronary heart disease (CHD), including coronary atherosclerosis and established coronary artery disease (CAD), to be around 47% in NAFLD patients [236]. A 2021 meta-analysis from Toh et al. demonstrated a pooled prevalence of CAD of 44.6% (95% CI: 36.0–53.6%) among 67,070 patients with NAFLD [237]. Moderate to severe steatosis had a higher prevalence of CHD compared to mild steatosis (37.5%, 95% CI: 15.0–67.2% vs. 29.6%, 95% CI: 13.1–54.0%) [238].

Myriad factors contribute to the association between CAD and NAFLD including dyslipidemias, systemic inflammation, insulin resistance, endothelial dysfunction, oxidative stress, and disturbances in gut microbiota [233,239]. NAFLD is also associated with increased arterial thickness, epicardial fat thickness, and presence of calcified and non-calcified coronary plaques, all contributing to CAD [240,241]. As mentioned, there seems to be a correlation between the degree of NAFLD and prevalence of CAD. One study showed that as the severity of NAFLD increases, the percentage of non-CAD patients decreases (*p* < 0.001) [242]. One way of defining the severity of CAD is using the Coronary Artery Diseases Reporting and Data System (CAD-RADS) [243]. Multiple studies have shown that more severe NAFLD was associated with multiple vessel coronary disease and more severe CAD scores [235,244,245,246,247].

While robust evidence connects NAFLD and CHD, the relationship between NAFLD and mortality is less clear. Several cohort studies and a 2016 meta-analysis from Targher et al. of 34,000 patients demonstrated that NAFLD was associated with an increase in all-cause mortality and fatal and/or non-fatal cardiovascular events [4,248,249,250]. However, several other studies have shown differing results. A cohort study in the United States using the NHANES III: 1988–1994 demonstrated that NAFLD was not associated with increased risk of all-cause mortality [251]. A 2016 meta-analysis by Wu et al. also did not show increased cardiovascular or all-cause mortality in NAFLD patients [34]. However, Wu et al. (2016) did show an increased incidence and prevalence of CVD in NAFLD patients, including CAD and atherosclerosis [34]. A large 2016 meta-analysis showed a non-significant pooled incidence rate ratio (IRR) for overall mortality between NAFLD and non-NAFLD patients of 1.05 (95% CI: 0.70–1.56) but a significantly increased adjusted hazard ratio for overall mortality in NAFLD patients of 1.04 (95% CI: 1.03–1.04) [2]. If only studies that identified NAFLD by imaging (versus imaging and serum enzymes) were included, the pooled IRR for cardiovascular mortality became significant with an IRR of 1.37 (95% CI: 1.23–1.54). Similarly, the relationship between NAFLD and acute MI is poorly characterized. Several cohort studies showed that NAFLD was associated with an increased risk of MI compared to non-NAFLD patients [252,253,254]. However, a cohort study from 2019 consisting of 18 million patients from Italy, the Netherlands, Spain, and the United Kingdom demonstrated that NAFLD was not associated with increased MI (HR of 1.01, 95% CI: 0.91–1.12) and stroke (HR: 1.04, 95% CI: 0.99–1.09) after controlling for covariates such as blood pressure, type 2 diabetes status, cholesterol level, statin use, and hypertension [255].

A 2020 meta-analysis including approximately 21 million patients identified NAFLD as an independent risk factor for acute coronary syndrome (ACS) in Asian populations, but this association conflicts with American and European studies [235]. Further, Ismaiel et al. characterized NAFLD as an independent predictor of all-cause and CV mortality as well as in-hospital major adverse cardiac events in ACS patients [235]. The impact of sex on NAFLD and CVD is poorly studied [239,256]. Female sex was associated with protection against ischemic cardiac events; however, female patients with NAFLD lose this benefit and experience increased CVD and mortality compared to men [257]. A 2020 meta-analysis by Khalid et al. demonstrated that in a population of 108,711 patients with NAFLD, with 44% being women with weighted mean age of 50 years, female sex was associated with increased all-cause mortality with odds ratio of 1.65 (95% CI: 1.12–2.43, *p* < 0.012) and increased CV events and mortality with OR of 2.12 (95% CI 1.65–2.73, *p* < 0.001) [24]. Meta-regression showed that women experienced higher mortality with advancing age beginning at age 42 [24]. Further studies are needed to elucidate the interplay of NAFLD, sex, CVD, and mortality.

Modifying risk factors remains critical for preventing and managing NAFLD [97,141,258]. Many of the same lifestyle modifications, such as diet, weight loss, and exercise, are also beneficial for managing CAD [259,260,261,262]. Weight loss of about 5% has been shown to decrease hepatic steatosis, while weight loss of around 10% is required to improve non-alcoholic steatohepatitis and fibrosis [140,141]. Bariatric surgery is known to have cardiovascular benefits and may also have a role in managing NAFLD [263,264,265]. Adherence to a calorie restricted diet can improve both NAFLD and atherogenesis [259,260,261]. Refs. [265,266,267,268,269] Particularly, a caloric reduction of at least 30% (750–1000 kcal/day) can improve both insulin resistance and hepatic steatosis. [27]. The MedDiet and DASH diet have both had positive effects in NAFLD patients, with the MedDiet improving hepatic steatosis [141,266,267,268,269]. Sedentary behavior increases mortality and may predispose patients to NAFLD [270,271]. While exercise has been shown to improve NAFLD, the specific parameters relating to the type of exercise and duration are unclear.

Ref. [239] Several studies, including the Pioglitazone versus Vitamin E versus Placebo for the Treatment of Nondiabetic Patients with Nonalcoholic Steatohepatitis (PIVENS) trial and a 2017 meta-analysis by Musso et al., showed that pioglitazone improved fibrosis even in non-diabetic patients with NASH [272,273,274]. Pioglitazone also has cardiovascular benefits, including reducing coronary disease, which may be beneficial in limiting mortality and cardiovascular events in NAFLD patients [275,276,277]. However, these medications have fallen to the background with the advent of GLP-1 agonists and SGLT-2 inhibitors. Liraglutide, a glucagon like peptide-1 analog, has been shown to reduce serum liver enzymes and fibrosis, improve steatosis, and reduce adverse cardiovascular events [278,279,280,281,282]. Vitamin E has also been suggested as a treatment for NAFLD given its anti-oxidative properties. Early studies of vitamin E were confounded by differing doses and formulations that could affect bioavailability [141]. However, vitamin E can improve serum liver enzymes, inflammation, and steatosis [141,283,284,285]. Previous concerns about increased all-cause mortality related to vitamin E have been unable to be reproduced in large meta-analyses [286,287,288]. Concerns remain about vitamin E and an increased risk of prostate cancer in limited patient populations [289,290,291].

Statins, which limit cholesterol biosynthesis through inhibition of 3-hydroxy-3-methylglutaryl coenzyme A reductase, have been shown to improve steatosis and NAFLD activity scores across multiple small studies, although robust, high-quality evidence is lacking, which limits their indication in managing liver disease in NAFLD [292,293,294,295,296,297]. Researchers have hypothesized that the beneficial effects of statins in NAFLD may in part be mediated through alterations in gut microbiota [298]. In contrast, statins have a definitive role in treating dyslipidemias and improving cardiovascular outcomes [227,228]. Despite some concern about statin use in patients with pre-existing liver disease, multiple studies have established the safety of statins in patients with NAFLD regardless of elevated baseline liver enzymes, and statins should be started in patients with NAFLD who meet criterion [299,300,301,302].

### 5.5. Heart Disease

Extensive evidence associates NAFLD with myocardial changes, including support for a graded relationship between functional and structural myocardial abnormalities and severity of NAFLD histology [303,304,305,306]. A cohort study of 181 patients showed that the prevalence of NAFLD in heart failure (HF) with preserved rejection fraction (HFpEF) may reach 50% [307]. Another cohort study of 102 patients found that the prevalence of NAFLD in heart failure with reduced ejection fraction (HFrEF) was 36.3% [308]. A Korean cohort study of 3300 patients by Chung et al. revealed that the prevalence of left ventricular (LV) diastolic dysfunction increased with NAFLD fibrosis grade (30.4% in non-NAFLD patients, 35.2% in NAFLD patients without advanced fibrosis, and 57.4% in NAFLD patients with advanced fibrosis, *p* < 0.001) [309].

Researchers have hypothesized that the relationship between NAFLD and structural cardiac changes stems primarily from systemic inflammation and other processes including insulin resistance, oxidative stresses, activation of the renin-angiotensin aldosterone system, and gut microbiota [70,310]. Inflammatory cytokines such as tumor necrosis factor-alpha (TNF-a), nuclear factor kappa-B (NF-kB), and interleukin-6 may result in inflammatory responses in myocardium and ventricular remodeling [310,311]. Multiple studies have confirmed these radiographic findings of cardiac dysfunction including increased myocardial, pericardial, and epicardial fat by magnetic resonance imaging and reduced early diastolic and systolic velocities in NAFLD patients [305,312]. In an analysis of patients from the multicenter, community-based Coronary Artery Risk Development in Young Adults (CARDIA) study, VanWagner et al. found that patients with NAFLD had lower early diastolic relaxation (e’) velocity (10.8 ± 2.6 vs. 11.9 ± 2.8 cm/s), higher LV filling pressure (E/e’ ratio: 7.7 ± 2.6 vs. 7.0 ± 2.3), and worse absolute GLS (14.2 ± 2.4% vs. 15.2 ± 2.4%) (*p* < 0.001 for all) suggestive of early subclinical LV systolic dysfunction [303]. Prior studies support the association between severity of NAFLD and severity of LV diastolic dysfunction [306,309,313]. Chung et al. demonstrated a significant incremental increase in risk of LV diastolic dysfunction in non-obese patients with NAFLD compared to patients without NAFLD (OR: 1.40, 95% CI: 1.06–1.84 for NAFLD without advanced fibrosis, OR: 1.44, 95% CI: 0.95–2.17 for NAFLD with advanced fibrosis, *p* = 0.022) [309].

Most investigations examining the relationship between NAFLD and HF have primarily combined HFpEF with HFrEF or looked at HFpEF alone, and strong evidence supports this association [307,308,314,315,316]. In a study of 870,535 Medicare beneficiaries, Fudim et al. found that the cumulative incidence and hazard ratio in Cox models were only significant for HFpEF, but not for HFrEF, when comparing patients with NAFLD vs. without NAFLD [317]. Recent studies have attempted to classify NAFLD and HFpEF into different subgroups through phenomapping, a clustering analysis that utilizes dense phenotypic data to identify phenotypically distinct classifications of HFpEF [318,319,320]. Salah et al. proposed three distinct phenotypes: obstructive HFpEF, metabolic HFpEF, and advanced liver disease/cirrhosis HFpEF (PMID: 34869957). Due to physiological differences between these phenotypes, additional investigation is required to further characterize mortality and optimal management of these phenotypic groups. A growing body of evidence supports the negative association between NAFLD and outcomes of patients with HF, including in-patient and post-discharge mortality as well as hospital readmissions [314,321,322,323]. In a cohort of elderly patients, Valbusa reported a 5-fold increase in 1-year all-cause re-hospitalization rate in NAFLD patients admitted for acute HF compared to non-NAFLD patients admitted for acute HF (HR: 5.05, 95% CI: 2.78–9.10, *p* < 0.001) [314]. In a large cohort study of about 3.5 million patients, Minhas et al. reported NAFLD was associated with higher mortality for both HFrEF and HFpEF (HFrEF, aOR: 1.84, 95% CI 1.66–2.04, *p* < 0.001, HFpEF, aOR: 1.65, 95% CI 1.43–1.9, *p* < 0.001) as well as increased length of stay and cost compared to non-NAFLD patients with HFrEF and HFpEF [323]. In an analysis of the SwedeHF registry, Ergatoudes showed that liver disease may be associated with worse outcomes in HFrEF compared to HFpEF (HR: 2.13, 95% CI 1.83–2.47 vs. HR 1.42, 95% CI 1.09–1.85, *p* = 0.02) [324]. This highlights the need for further work comparing the impact of NAFLD on HFrEF and HFpEF.

Lifestyle management remains an integral part of preventing and treating NAFLD and HF [140,141,325,326]. As discussed, weight loss has been shown to improve NAFLD, and prior studies demonstrated that weight loss ameliorated myocardial structural changes in obesity [140,141,327]. Therefore, weight loss of at least 5% is recommended in patients with NAFLD for hepatic and cardiac benefits [140,141]. Weight reduction via diet or bariatric surgery was shown to have these benefits [328,329,330,331]. Another benefit of bariatric surgery is that it may be used as a bridge for weight loss in morbidly obese patients ineligible for cardiac transplantation [332]. The visceral fat deposits commonly seen in obese patients may be resistant to mild changes in diet-induced weight reductions [316,331]. In a small randomized controlled trial studying dietary interventions in obese subjects, de las Fuentes observed partial weight regain at 24 months (average percent weight loss at 3: 7.3 ± 4.0%, 6: 9.2 ± 5.6%, 12: 7.8 ± 6.6%, 24: 3.8 ± 7.9% months) [331]. Although the maximal beneficial effects of weight loss were reduced, structural cardiovascular parameters still showed significant improvement compared to baseline [331]. From a dietary perspective, it is recommended that NAFLD patients adhere to a hypocaloric diet with a daily consumption of 1200–1800 kcal or daily deficit of 500–1000 kcal from baseline [140,333,334]. The DASH diet and MedDiet have been shown to improve NAFLD and may improve cardiovascular risks as well [146,269]. The MedDiet is thought to modulate hepatic and cardiovascular risks through alterations in insulin resistance, serum triglyceride levels, intrahepatic triglyceride levels, and serum cholesterol levels [147,149]. Given these benefits, multiple societies recommend the MedDiet for patients with NAFLD [140,333,334]. As discussed, exercise offers numerous benefits including decreased intrahepatic fat, improved serum metabolic markers, and cardiac function parameters such as early diastolic filling [335,336]. Several studies have shown no difference in aerobic vs. resistance training in reducing intrahepatic fat content in NAFLD patients [337,338,339]. However, a 2021 meta-analysis by Xiong et al. showed that aerobic exercise reduced more metabolic indicators compared to resistance exercise and high-intensity interval training in NAFLD patients specifically [340]. Additionally, robust evidence supports the association between the aggressiveness of weight loss intervention and improvement in NAFLD [268,341,342,343]. A paradoxical relationship exists between obesity and cardiac dysfunction [326]. When patients have clinically symptomatic HF, higher BMI has a protective mortality benefit [344,345,346,347]. Thus, patients with advanced cardiac dysfunction must be cautious before beginning weight loss regimens.

Multiple medications have been investigated for treating NAFLD and the ability to concomitantly manage HF symptoms. Pioglitazone was previously mentioned as having beneficial effects related to ischemic CVD; however, pioglitazone can lead to weight gain and increased peripheral edema in a dose-dependent fashion in approximately 5–10% of patients [348,349]. The concern for fluid retention and possible triggering of HF limits the use of pioglitazone in these patients. GLP1-receptor agonists are rapidly becoming of interest, given weight loss benefits and improved insulin resistance. In several studies, including the Liraglutide Effect and Action in Diabetes (LEAD) program, LEAD-2 study, and Liraglutide Efficacy and Action in NASH (LEAN) trial, liraglutide improved hepatic steatosis and resolution of NASH in biochemistry-based or in biochemistry-defined, imaging-defined, and biopsy-defined NAFLD, although liraglutide failed to improve hepatic fibrosis [279,280,350,351]. Semaglutide, another GLP-1 receptor agonist, has also shown promising results in several trials [350]. A 2019 meta-analysis of eight studies by Kristensen et al. demonstrated that GLP-1 receptor agonists reduced hospitalizations for heart failure by 9% [281]. This evidence supports the use of these medications in managing diabetes in patients with comorbid heart failure. SGLT2-inhibitors have also demonstrated extraordinary promise in NAFLD patients with heart failure. Multiple meta-analyses have demonstrated that SGLT2-inhibitors improved liver function tests and hepatic fat as assessed by imaging techniques [280,352,353,354]. Many meta-analyses and trials, including the Empagliflozin Outcome Trial in Patients With Chronic Heart Failure With Reduced Ejection Fraction (EMPEROR-Reduced), Empagliflozin Outcome Trial in Patients With Chronic Heart Failure With Preserved Ejection Fraction (EMPEROR-Preserved), and Dapagliflozin and Prevention of Adverse Outcomes in Heart Failure (DAPA-HF), showed that SGLT2 inhibitors can decrease hospitalization for HF in both HFrEF and HFpEF patients regardless of diabetes status [355,356,357,358]. A 2021 meta-analysis by Salah et al. quantified this effect and demonstrated a 31% reduction in hospitalization for HF in patients taking SGLT2 inhibitors [359].

Novel agents that can treat NAFLD and reverse fibrosis continue to be developed. Four medications are currently farthest along in development, undergoing phase III trials [158,326,360]. Current evidence does not reveal any impact of obetacholic acid on HF [158,239]. Elafibranor, a peroxisome proliferator−activated receptor-alpha and peroxisome proliferator−activated receptor-delta agonist, improves steatohepatitis without worsening fibrosis [360]. Elafibranor did not cause weight gain or increase cardiac events [360]. Cenicrivoric, a dual chemokine receptor (CCR) 2 and 5 antagonist, has been shown to improve liver fibrosis without worsening steatohepatitis [162]. While the trial did not report cardiovascular benefits, prior studies using mouse models have shown CCR2 blockade may decrease atherosclerotic lesion development, possibly portending cardiovascular benefit in humans [162,361]. Selonsertib is an apoptosis signal-regulating kinase 1 (ASK1) inhibitor that has also been shown to improve liver fibrosis [362]. This trial also did not report cardiovascular benefits, but ASK1 is known to affect the cellular stress response and apoptosis pathway, contributing to diseases such as heart failure and ischemia/reperfusion injury [362,363,364,365,366]. ASK1 deficient mice displayed reduced levels of cardiomyocyte apoptosis, hypertrophy, and interstitial fibrosis [367]. Thus, although further investigation is needed, ASK1 inhibitors represent an ideal class of medications for NAFLD patients with HF. The above subsections are summarized here below in Table 2.

## 6. Conclusions

This narrative review aims to provide a contemporary understanding of NAFLD and its associations with cardiovascular disease, while highlighting strategies to combat the rising prevalence of both conditions, each of which confers notable morbidity and mortality. Both inherent and preventable risk factors for NAFLD pathogenesis largely parallel those for cardiovascular disease. Accordingly, a variety of studies have demonstrated associations between NAFLD and various CVD states including CHF, MI, cardiac arrhythmias, and CAD. While NAFLD itself is not implicated in CVD pathophysiology, various biochemical and physiological factors ranging from systemic inflammation, endothelial dysfunction, dyslipidemia, and altered glucose metabolism can precipitate both conditions. Heritable risk factors have also been recently elucidated in studies comparing NAFLD rates between races or within families to analyze genetic susceptibility to certain metabolic abnormalities. Although NAFLD treatment remains elusive, the close relationship between this condition and CVD suggests that standard CVD management such as antihypertensives, diabetes medications, and relevant lifestyle modifications may prove advisable. An improved understanding of the relationships between NAFLD and CVD can improve patient counseling, management, and public health programming to reduce overall disease burden.

## Figures and Tables

**Table 1:** Overall NAFLD cardiac complications.

Overall NAFLD Cardiac Complications	
General ACSVD Scores	Meta-analyses suggest higher degrees of NAFLD are associated with higher ACSVD scores (34–36)
Heart Failure	Meta-analyses demonstrate that NAFLD is associated with higher odds ratios for developing diastolic dysfunction (43–44)
Myocardial Infarction	Meta-analyses show NAFLD is associated with increased odds ratio for developing a myocardial infarction (44, 46–48)
Cardiac Arrhythmias	Meta-analyses demonstrate NAFLD is associated with higher risk of developing cardiac arrhythmias, most notably atrial fibrillation (44, 53–55)
Coronary Artery Disease	Epidemiologic data shows that patients with NAFLD have higher rates of cardiovascular events and development of coronary artery plaques (4, 56–58)

**Table 2:** Summary of Co-morbidities in NAFLD and preventives to lower ASCVD.

Cardiovascular Co-morbidities in NAFLD and Preventive Strategies to Lower ASCVD	
Hypertension	Lifestyle modifications, RAAS inhibitors, selected agents in clinical trials (Obeticholic acid, Pegbelfermin, Elafibranor)
Diabetes Mellitus	Lifestyle modifications, metformin, GLP-1 agonists, DPP-4 inhibitors
Metabolic Syndrome	Lifestyle modifications, fibrates, aldosterone receptor blockers, bariatric surgery
Coronary Artery Disease	Lifestyle modifications, pioglitazone, statins, bariatric surgery
Heart Disease	Lifestyle modifications, GLP-1 receptor agonists, selected agents in clinical trials (Obeticholic acid, Elafibranor, Cenicrivoric, Selonsertib)
